# Development of a predictive model for histological chorioamnionitis in patients with preterm premature rupture of membranes: a single-center retrospective cohort study

**DOI:** 10.7717/peerj.21929

**Published:** 2026-11-12

**Authors:** Jindi Zhang, Hanglin Wu, Fangyuan Zheng, Xia Liu, Yun Wang

**Affiliations:** 1 Department of Obstetrics, Hangzhou Women’s Hospital (Hangzhou Maternity and Child Health Care Hospital), Hangzhou, Zhejiang Province, China; 2 Department of Nursing, Hangzhou Women’s Hospital (Hangzhou Maternity and Child Health Care Hospital), Hangzhou, Zhejiang Province, China

**Keywords:** Vaginal examinations, Preterm premature rupture of membrane, Histological chorioamnionitis, Prediction model, TRIPOD

## Abstract

**Objective:**

The aim of this study is to explore the risk factors for histological chorioamnionitis (HC) and to develop a preliminary clinical prediction model among women with preterm premature rupture of membranes (PPROM) during inpatient expectant management.

**Methods:**

This single-center retrospective cohort study consecutively enrolled women with singleton PPROM at 28 + 0 ∼ 36 + 6 weeks of gestation who delivered at Hangzhou Women’s Hospital between December 2022 and October 2025. Clinical and laboratory data recorded from admission to delivery were extracted. Univariate analysis was followed by multivariable logistic regression. Candidate predictors were selected on clinical plausibility and statistical significance. A binary logistic regression model was fitted, and apparent discriminative performance was summarized with a receiver operating characteristic (ROC) curve.

**Results:**

A total of 306 women were included (HC group, *n* = 152; control group, *n* = 154). In the multivariable model, vaginal examinations (VE), antibiotic beginning time (ABT), reproductive tract pathogens (RTP), white blood cell count (WBC), and C-reactive protein (CRP) remained independently associated with HC. The fitted model was *z* = 1.874 × ln(VE) + 1.051 × ln(ABT) + 2.378 × ln(WBC) + 0.821 × ln(CRP) + 0.846 × RTP (0 or 1) −11.054. The apparent area under the ROC curve (AUC) was 0.901 (95% confidence interval (CI) [0.864–0.934]); the optimism-corrected AUC after bootstrap internal validation was 0.894, and calibration was adequate (Hosmer–Lemeshow *P* = 0.409; Brier score 0.128).

**Conclusions:**

VE, ABT, RTP, WBC, and CRP were identified as independent risk factors for HC in patients with PPROM. The resulting model is a preliminary risk-stratification tool intended for use during inpatient management. It requires external validation in independent populations and an admission-only predictor version before clinical implementation. It is not intended for prediction at the time of admission.

## Introduction

Preterm premature rupture of membranes (PPROM) is one of the leading causes of preterm birth, and complicates approximately 2–4% of pregnancies globally (Geremew et al., 2024). It substantially elevates the risk of neonatal complications, including infection, respiratory distress syndrome, and cerebral palsy (Guo et al., 2023). Histologic chorioamnionitis (HC) frequently complicates PPROM and is strongly associated with adverse maternal and neonatal outcomes, such as sepsis and neonatal encephalopathy (Sotodate et al., 2025).

PPROM is defined as spontaneous rupture of the fetal membranes prior to the onset of labor in pregnancies before 37 weeks of gestation, with a multifactorial etiology, involving several pathological mechanisms that may act independently or synergistically (Ushida et al., 2024; Quiñones Rivera et al., 2025). Chorioamnionitis represents a common obstetric complication, characterized by a reciprocal causal relationship with PPROM (Panneflek et al., 2024). Ascending infection from the vagina and cervix is the most prevalent route of microbial invasion  (Balogh et al., 2025). Following membrane rupture, the uterine cavity becomes exposed to the external environment, allowing bacteria to ascend and trigger a local inflammatory response. This leads to the release of abundant chemokines and inflammatory mediators, prompting massive neutrophil migration into the chorion and amnion, and resulting in HC (Lanzarone et al., 2025; Kay et al., 2024). Furthermore, infection by various microorganisms markedly increases intrauterine levels of endotoxins, cytokines (*e.g.*, TNF-*α*), matrix metalloproteinases, and prostaglandins (Daskalakis et al., 2023). These factors promote degradation of the extracellular matrix, cervical remodeling, loss of membrane integrity, uterine contractions, and ultimately membrane rupture (Nguyen, Aronoff & Eastman, 2023; Takahashi et al., 2025). Both PPROM and HC are associated with elevated risks of adverse maternal and fetal outcomes, including umbilical cord prolapse, fetal distress, neonatal infection, and preterm delivery (Gilboa et al., 2025).

Current research primarily focuses on clinical chorioamnionitis, which is characterized by overt signs such as fever and leukocytosis. In contrast, HC poses diagnostic challenges due to its frequently asymptomatic nature and the requirement of histopathological confirmation post-delivery. This often leads to delayed diagnosis and missed opportunities for timely intervention, thereby increasing the risk of adverse pregnancy outcomes (Cornet et al., 2025). Moreover, the risk factors for HC remain incompletely elucidated, and the scarcity of quantitative studies on dynamic biomarkers has hindered the development of reliable predictive tools for clinical use.

The present study was designed to identify risk factors for HC and to develop a preliminary prediction model among women with PPROM managed expectantly in hospital. Because several predictors were recorded cumulatively until delivery, the model is intended as an aid to risk stratification during inpatient management rather than as a tool for prediction at the time of initial presentation.

## Materials & Methods

### Study design and setting

This single-center retrospective cohort study included pregnant women with PPROM who were hospitalized and delivered at Hangzhou Women’s Hospital between December 2022 and October 2025. Consecutive enrollment was used to limit selection bias. The study was approved by the Ethics Committee of Hangzhou Women’s Hospital (Medical Ethics Review No. 2025-121). Because the study was retrospective and used only de-identified clinical and laboratory data, the ethics committee exempted the requirement for informed consent. All patient data were kept strictly confidential. The study was conducted in accordance with the Declaration of Helsinki.

### Participants

Women with singleton pregnancies complicated by PPROM at 28 +0∼36 +6 weeks of gestation were eligible. PPROM was defined as spontaneous rupture of membranes before the onset of labor and before 37 completed weeks of gestation, confirmed by a combination of history, sterile speculum examination showing pooling of amniotic fluid, and/or a positive nitrazine test. We excluded women with: (i) severe pregnancy complications (*e.g.*, severe preeclampsia, severe intrahepatic cholestasis of pregnancy, severe autoimmune disease, cardiovascular disease, liver or kidney disease, malignant tumors, severe infection); (ii) missing clinical data, including laboratory results, prenatal records, or placental pathology; (iii) fetal death in utero; (iv) cervical cerclage before membrane rupture; (v) multiple pregnancy. The final analytic sample comprised 306 women.

### Outcome

The outcome was HC, diagnosed according to the 2016 Amsterdam Placental Workshop Group criteria (Khong et al., 2016). Pathological slides were first assessed by a pathologist with more than 5 years of experience and then independently reviewed by a senior pathologist with more than 10 years of experience. Both pathologists were blinded to clinical data. Inter-observer agreement between the two pathologists for the diagnosis of HC was substantial (Cohen’s *κ* = 0.73). Discrepancies were resolved by joint review. HC was considered present when neutrophilic infiltration involved the chorion and/or amnion (stage 1 or higher) and/or the chorionic plate (maternal inflammatory response) or the umbilical cord (fetal inflammatory response), as defined by the Amsterdam criteria.

### Predictors and measurements

The following variables were collected from electronic medical records: maternal age, gravidity, parity, body mass index (BMI) at admission, gestational weeks (GW) at membrane rupture, time from membrane rupture to delivery (Duration), frequency of vaginal examinations (VE), antibiotic beginning time (ABT), administration of antenatal glucocorticoids (GC), use of labor analgesia (LA), highest recorded temperature (T), highest white blood cell count (WBC), and highest C-reactive protein (CRP) during the observation period from PPROM onset to delivery. VE was defined as the total number of speculum and digital vaginal examinations performed from membrane rupture to delivery. ABT was defined as the interval (hours) from PPROM to initiation of antibiotic therapy. Temperature, WBC, and CRP were recorded as the peak values observed during inpatient observation.

Peripheral venous blood samples were collected into EDTA tubes. Complete blood count was measured with an automated five-part differential hematology analyzer (Sysmex XN-2000; Kobe, Japan). CRP was measured on the same Sysmex XN-2000 automated hematology analyzer equipped with an immunoturbidimetric detection module, enabling simultaneous whole-blood CRP measurement together with the complete blood count. The laboratory assays were performed by certified technicians according to the manufacturer’s standardized operating procedures. Results were reported in conventional units (WBC, $\times 10\hat {}$ 9/L; CRP, mg/L; temperature, °C).

### Microbiological methods

Reproductive tract secretion specimens were obtained on admission using a standardized sterile speculum technique: the external genitalia were disinfected with iodine, the cervix was exposed with a sterile speculum, the external cervical os was wiped with a sterile cotton ball, and a sterile swab was inserted approximately one cm into the cervical canal, rotated 3–5 times, and left in place for at least 10 s. Specimens were transported in Amies transport medium and cultured within 2 h. Culture panels included *Mycoplasma hominis*, *Ureaplasma urealyticum*, *Escherichia coli*, *Klebsiella pneumoniae*, *group B Streptococcus*, *Enterococcus faecalis*, *Streptococcus pyogenes*, and *Staphylococcus aureus*. Cultures were considered positive if any pathogen was isolated; mixed growth was recorded as positive. Cultures obtained after the initiation of antibiotics were excluded from analysis. The reproductive tract pathogens (RTPs) variable was coded as 1 if any of the above organisms was isolated and 0 if all cultures were negative.

### Statistical analysis

Data were analyzed with SPSS (version 27.0, IBM Corp., Armonk, NY, USA). The Shapiro–Wilk test was used to assess normality. Most continuous variables were skewed and are reported as median (25th–75th percentile). Between-group comparisons were performed with the Mann–Whitney *U* test for continuous variables and the chi-square test for categorical variables. Univariate logistic regression was used to estimate crude odds ratios (ORs) with 95% confidence intervals (CIs).

Collinearity among the candidate predictors was assessed with variance inflation factors and pairwise Spearman rank correlations. Candidate predictors for the multivariable model were selected on the basis of clinical relevance and univariate association (*P* < 0.05). Because several variables were highly skewed, significant continuous predictors were natural-log transformed before inclusion in the final binary logistic regression. The final model retained predictors that remained statistically significant after adjustment and were considered clinically interpretable.

The cohort was assembled retrospectively from consecutive admissions over a fixed period, so no formal sample size calculation was performed. Model performance was summarized with the area under the receiver operating characteristic curve (AUC) and its 95% CI. Events per variable (EPV) are reported for both the exploratory and the final model, and the potential for overfitting was further examined by bootstrap validation. All tests were two-sided, and *P* < 0.05 was considered statistically significant. Calibration was assessed by a calibration plot of predicted *versus* observed probabilities across deciles of predicted risk, the Hosmer–Lemeshow test, the Brier score, and the bootstrap-validated calibration slope.

### Reporting

This manuscript was prepared according to the TRIPOD+AI statement for reporting clinical prediction models (Anon, 2024). A completed TRIPOD checklist is provided as a Document S1.

## Results

### Participant flow and baseline characteristics

During the study period, 14,538 women delivered at Hangzhou Women’s Hospital. Of these, 1,056 had PPROM. After applying the predefined eligibility criteria, 306 women were included: 152 in the HC group and 154 in the control group (Fig. 1).

**Figure 1:**
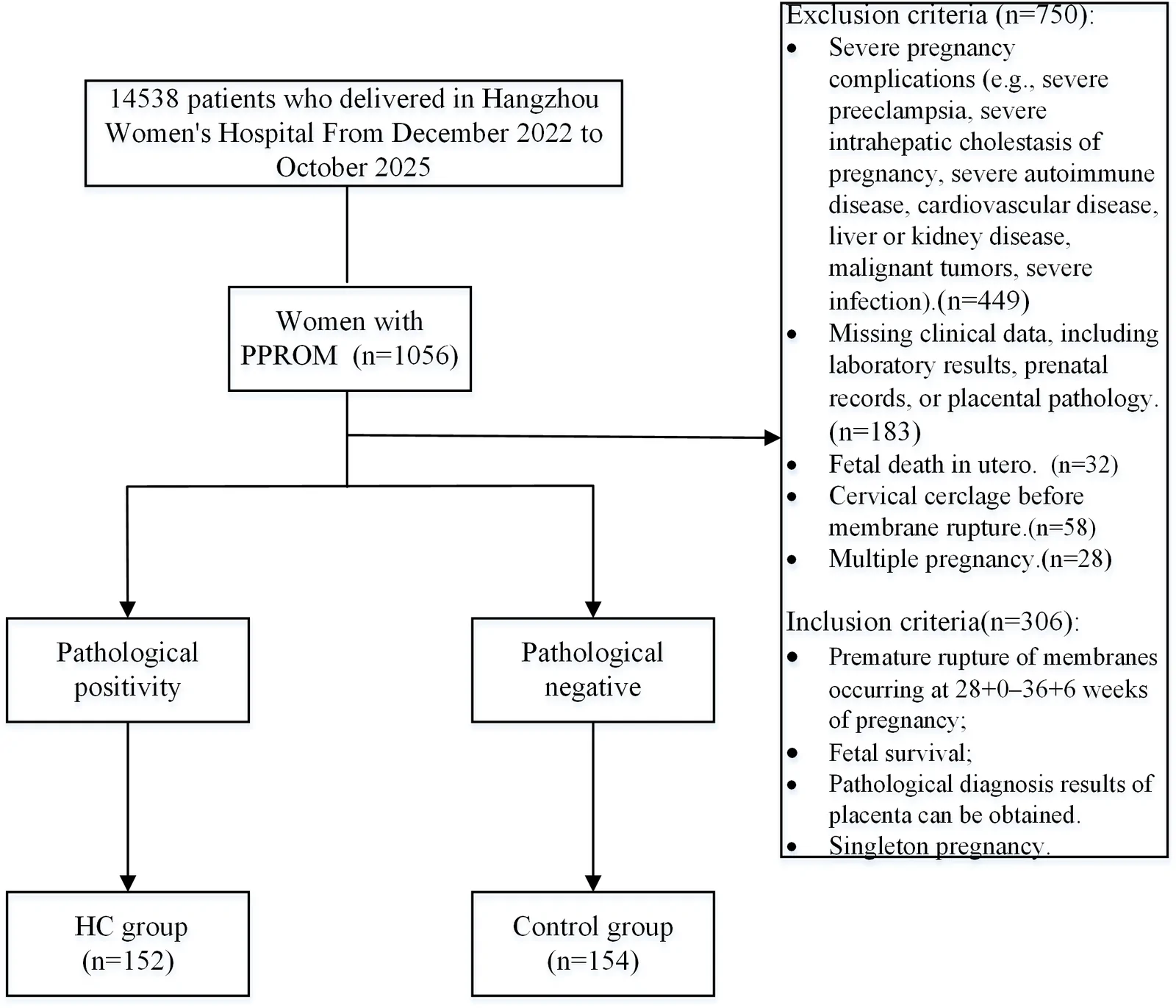
Flow diagram of participant recruitment for the development cohort (December 2022–October 2025). PPROM, preterm premature rupture of membranes; HC, histological chorioamnionitis.

Of the 306 women enrolled, CRP was missing for 12 (3.9%) and ABT for 3 (1.0%); all other variables were complete, and the 15 women with missing values were excluded listwise, leaving 291 complete cases (147 with histological chorioamnionitis, 144 without) for all regression models (Table S1). The final model therefore rests on 147 events against five predictor degrees of freedom (EPV 29.4), which is comfortable by conventional standards; the exploratory model that screened all 14 candidate predictors had an EPV of 10.5, above the customary minimum of 10 but with little margin. Bootstrap validation returned an optimism of 0.007 in the AUC, indicating that overfitting was limited.

There were no statistically significant differences between groups in BMI, gravidity, parity, or LA. Statistically significant differences were observed for age, gestational weeks at rupture, duration of membrane rupture, VE, ABT, RTP, glucocorticoid administration, temperature, WBC, and CRP (Table 1).

**Table 1:** Univariate analysis of clinical data between two groups.

Indicators	Control group (*n* = 154)	HC group (*n* = 152)	*Z*/*χ*2	*P*
Age (years)	30.00 (28.00–33.00)	31.00 (29.00–34.00)	2.030	0.042^*^
BMI (Kg/m^2^)	26.04 (23.38–27.93)	25.81 (23.69–28.04)	0.547	0.585
Gravidity			0.204	0.652
=1	83 (53.90)	78 (51.32)		
≧2	71 (46.10)	74 (48.68)		
Parity			1.765	0.184
=0	114 (74.03)	102 (67.10)		
≧1	40 (25.97)	50 (32.90)		
GW (weeks)	35.00 (33.00–35.00)	33.00 (30.00–34.00)	−6.785	<0.001^**^
Duration (hours)	13.00 (6.00–31.50)	78.00 (26.75–156.00)	9.661	<0.001^**^
VE	4.00 (2.00–6.00)	7.00 (4.00–9.00)	7.600	<0.001^**^
ABT (hours)	2.00 (1.00–3.00)	3.00 (2.00–6.00)	5.002	<0.001^**^
RTP			14.231	<0.001^**^
=0	94 (61.04)	60 (39.47)		
=1	60 (38.96)	92 (60.53)		
GC			29.192	<0.001^**^
=0	94 (61.04)	46 (30.26)		
=1	60 (38.96)	106 (69.74)		
LA			0.765	0.382
=0	114 (74.03)	119 (78.29)		
=1	40 (25.97)	33 (21.71)		
T (°C)	37.20 (37.10–37.30)	37.40 (37.20–37.80)	6.155	<0.001^**^
WBC ($\times 10\hat {}$^9^/L)	8.80 (7.50–10.30)	11.80 (9.50–15.30)	7.781	<0.001^**^
CRP (mg/L)	2.70 (1.56–5.29)	7.72 (2.92–20.79)	6.985	<0.001^**^

**Notes.**

The quantitative data were expressed by median and percentile [M (P_25_–P_75_)], and qualitative data were expressed by *n* (%).

* *P* < 0.05.

** *P* < 0.001.

HC Histologic chorioamnionitis BMI Body mass index GW Gestational weeks VE Vaginal examinations ABT Antibiotic beginning time RTP Reproductive tract pathogens GC glucocorticoids LA Labor analgesia T Temperature WBC White blood cell CRP C-Reactive protein

CRP was missing for 12 women and ABT for 3 women; descriptive statistics for these two variables are based on available cases.

The most frequently isolated organisms were *Mycoplasma* species, *Escherichia coli*, *Klebsiella pneumoniae*, *group B Streptococcus*, *Enterococcus faecalis*, *Streptococcus pyogenes*, and *Staphylococcus aureus*. *Mycoplasma* species were the most prevalent, identified in 127 women overall.

### Exploratory multivariable analysis

Exploratory multivariable logistic regression (Table 2) showed that parity, VE, ABT, RTP, LA, WBC, and CRP were statistically significant. GW and duration of membrane rupture were significant in univariate analysis but did not remain significant after adjustment.

**Table 2:** Exploratory multivariable logistic regression of clinical variables associated with HC.

Indicators	B	SE	Wald	df	*P*	OR (95% CI)
Age	0.067	0.057	1.399	1	0.237	1.069 (0.957–1.195)
Gravidity	−0.138	0.561	0.060	1	0.806	0.871 (0.290–2.618)
Parity	1.535	0.627	5.981	1	0.015^*^	4.639 (1.356–15.868)
BMI	−0.020	0.061	0.111	1	0.739	0.980 (0.870–1.104)
GW	−0.094	0.081	1.363	1	0.243	0.910 (0.777–1.066)
Duration	0.003	0.002	1.432	1	0.232	1.003 (0.998–1.008)
VE	0.595	0.102	33.760	1	<0.001^**^	1.814 (1.484–2.217)
ABT	0.258	0.081	10.173	1	0.001^*^	1.294 (1.105–1.517)
RTP	1.458	0.448	10.589	1	0.001^*^	4.295 (1.785–10.333)
GC	0.588	0.471	1.560	1	0.212	1.800 (0.716–4.530)
LA	−1.468	0.554	7.013	1	0.008^*^	0.230 (0.078–0.683)
T	0.647	0.690	0.879	1	0.349	1.910 (0.494–7.387)
WBC	0.232	0.087	7.077	1	0.008^*^	1.261 (1.063–1.497)
CRP	0.054	0.027	4.111	1	0.043^*^	1.056 (1.002–1.112)

**Notes.**

Multivariable binary logistic regression including all 14 candidate predictors, fitted on the complete-case dataset (*n* = 291; 147 events; events-per-variable = 10.5).

BMI Body mass index GW Gestational weeks VE Vaginal examinations ABT Antibiotic beginning time RTP Reproductive tract pathogens GC glucocorticoids LA Labor analgesia T Temperature WBC White blood cell CRP C-Reactive protein CI confidence interval

* *P* < 0.05.

** *P* < 0.001.

No collinearity that would distort the models was apparent: variance inflation factors ranged from 1.06 to 2.13, and the largest Spearman correlation involving parity or LA was that between parity and gravidity (*ρ* = 0.682), followed by LA and the number of VE (*ρ* = 0.423) (Table S2).

### Final prediction model

After natural-log transformation of the continuous predictors and selection of the most parsimonious, clinically coherent set, the final model retained VE, ABT, RTP, WBC, and CRP (Table 3). The model equation was:

**Table 3:** Binary logistic regression model after natural-log transformation of continuous predictors.

Indicators	B	SE	Wald	df	*P*	OR (95% CI)
VE	1.874	0.346	29.322	1	<0.001^**^	6.513 (3.305–12.833)
ABT	1.051	0.231	20.668	1	<0.001^**^	2.860 (1.818–4.499)
RTP	0.846	0.333	6.447	1	0.011^*^	2.330 (1.213–4.475)
WBC	2.378	0.697	11.630	1	<0.001^**^	10.785 (2.749–42.306)
CRP	0.821	0.181	20.586	1	<0.001^**^	2.272 (1.594–3.239)
Constant	−11.054	1.724	41.113	1	<0.001^**^	0

**Notes.**

Final logistic regression model after natural-logarithm (ln) transformation of the continuous predictors, fitted on the complete-case dataset (*n* = 291; 147 events; events-per-variable = 29.4).

VE Vaginal examinations ABT Antibiotic beginning time RTP Reproductive tract pathogens WBC White blood cell CRP C-Reactive protein CI confidence interval

* *P* < 0.05.

** *P* < 0.001.

*z* = 1.874 × ln(VE) +1.051 × ln(ABT) +2.378 × ln(WBC) +0.821 × ln(CRP) +0.846 × RTP (0 or 1) − 11.054.

The predicted probability of HC was calculated as *P* = 1/(1 +e$\hat {}-z$).

### Model performance

The apparent discriminative performance of the final model, evaluated in the same sample used for model development, yielded an AUC of 0.901 (95% CI [0.864–0.934]) (Fig. 2). At the optimal cut-off of 0.450, the model achieved a sensitivity of 85.0% (95% CI [78.4%–89.9%]), a specificity of 79.9% (95% CI [72.6%–85.6%]), and a Youden index of 0.649 (95% CI [0.592–0.760]) (Table 4).

**Figure 2:**
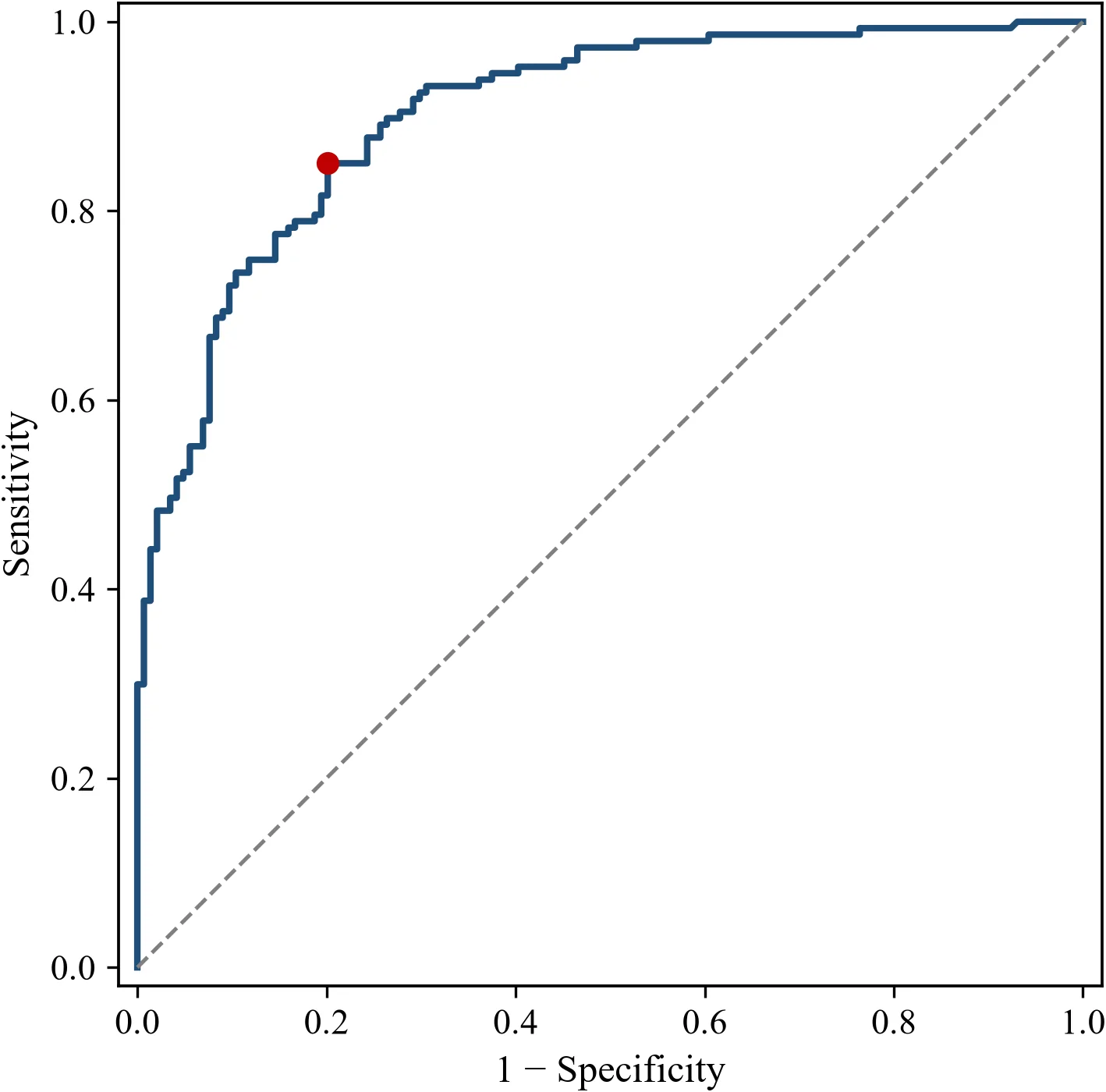
Receiver operating characteristic (ROC) curve of the final prediction model in the development sample.

**Table 4:** Performance of the final model for predicting HC.

Indicator	Value	95% CI
Youden index	0.649	0.592–0.760
Cut-off probability (Youden-optimal)	0.450	–
Sensitivity	85.0%	78.4–89.9%
Specificity	79.9%	72.6–85.6%
Positive predictive value (PPV)	81.2%	74.3–86.6%
Negative predictive value (NPV)	83.9%	76.9–89.1%
Positive likelihood ratio (LR+)	4.22	3.03–5.89
Negative likelihood ratio (LR−)	0.19	0.13–0.28
AUC	0.901	0.864–0.934

**Notes.**

Classification at cut-off 0.450: true positives 125, false positives 29, true negatives 115, false negatives 22. CIs for sensitivity, specificity, PPV and NPV are Wilson score intervals; CIs for LR+ and LR− were computed on the log scale; the 95% CI for the Youden index was estimated by non-parametric bootstrap with 5,000 resamples, refitting the model in each resample. AUC, area under the receiver operating characteristic curve.

CI confidence interval LR likelihood ratio

The calibration of the model was assessed graphically and statistically (Fig. 3). The Hosmer–Lemeshow test across 10 groups was not significant (*χ*^2^ = 8.26, *P* = 0.409), the Brier score was 0.128, and the bootstrap-validated calibration slope was 0.944, indicating adequate agreement between predicted probabilities and observed outcomes.

**Figure 3:**
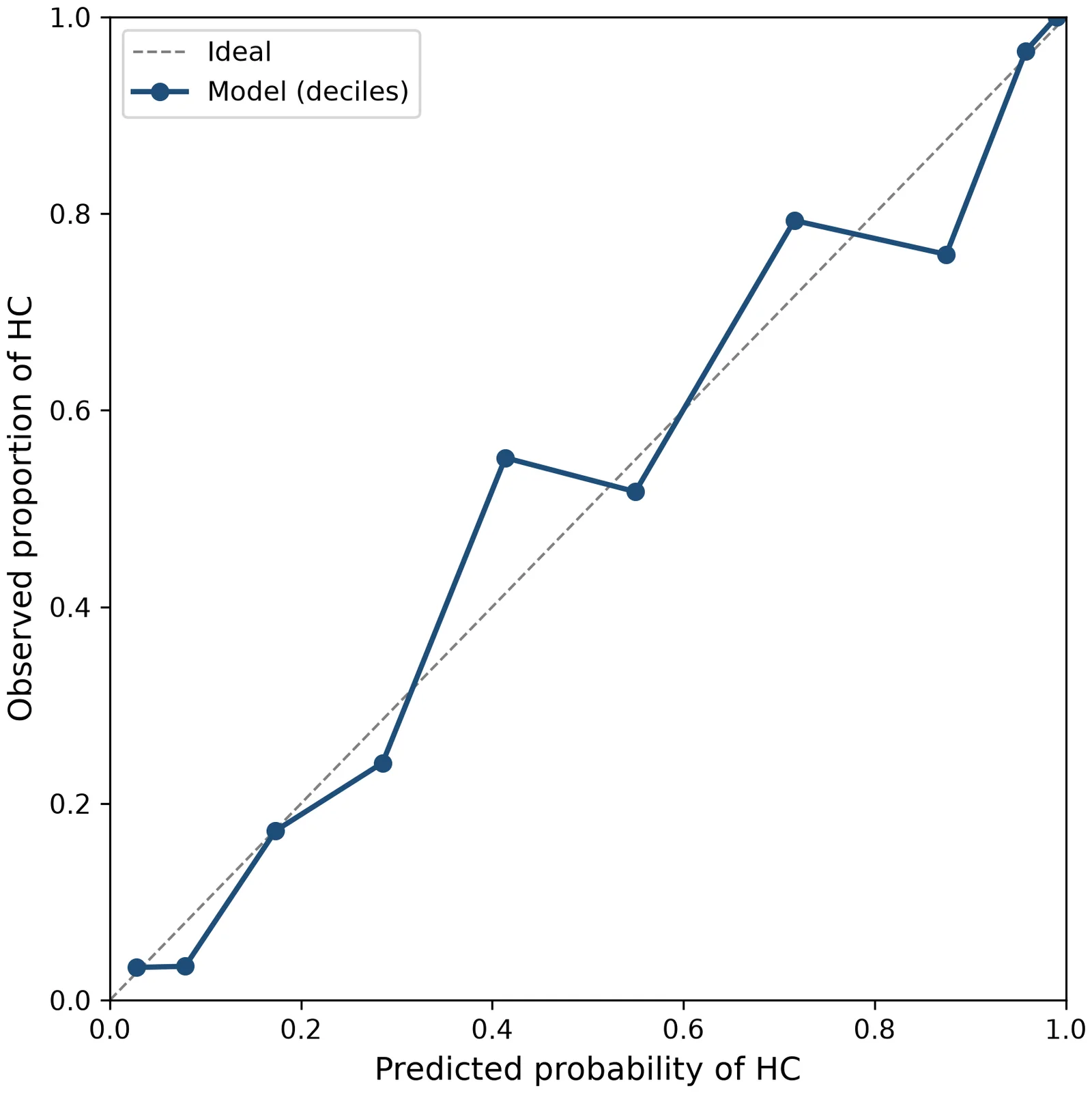
Calibration plot of the final five-variable model. Predicted probabilities are plotted against observed outcomes across deciles of predicted risk (*n* = 291). The diagonal line represents perfect calibration. The Hosmer–Lemeshow test across 10 groups was not significant (*χ*^2^ = 8.26, *P* = 0.409); Brier score = 0.128; bootstrap-validated calibration slope = 0.944.

### Sensitivity analyses

Adding parity and LA to the five final predictors left the core variables essentially undisturbed: ln(VE), ln(ABT), ln(WBC), ln(CRP) and RTP all retained their direction and statistical significance, while parity (OR 2.94, 95% CI [1.27–6.82]) and LA (OR 0.20, 95% CI [0.08–0.51]) reproduced the associations seen in the exploratory model. The apparent AUC rose from 0.901 to 0.918. Since the gain in discrimination was slight and came at the cost of two variables whose estimates are difficult to interpret causally, we retained the five-variable model (Table S3).

## Discussion

This single-center retrospective cohort study identified VE, ABT, RTP, WBC, and CRP as independent risk factors for HC in women with PPROM and developed a corresponding logistic regression model. Because several predictors were recorded as cumulative or peak values over the interval from membrane rupture to delivery, the model is best viewed as a risk-stratification tool for use during inpatient expectant management rather than an early prediction model at the time of first presentation.

The apparent AUC of the final model was 0.901 (95% CI [0.864–0.934]). Bootstrap validation with 1,000 resamples returned an optimism of only 0.007, giving a corrected AUC of 0.894, which indicates that the degree of overfitting is small. Calibration was likewise adequate: the Hosmer–Lemeshow test was not significant (*P* = 0.409), the Brier score was 0.128, and the bootstrap calibration slope was 0.944, close to the ideal value of 1. At the optimal cut-off of 0.450, the model achieved a sensitivity of 85.0%, a specificity of 79.9%, a positive likelihood ratio of 4.22 and a negative likelihood ratio of 0.19; the corresponding Youden index was 0.649 (95% CI [0.592–0.760]). A negative likelihood ratio below 0.2 means that a low predicted probability usefully lowers the suspicion of HC, which is the more actionable direction for a triage instrument of this kind.

A fundamental limitation of this model is chronological (look-ahead) bias. Four of the five predictors—the total number of vaginal examinations, the interval from membrane rupture to antibiotic initiation, and the peak WBC and CRP values—accumulate information over the entire course of expectant management up to delivery. Consequently, the model cannot be applied at the time of admission, when these quantities are still unknown, and its reported performance (apparent AUC 0.901, optimism-corrected 0.894) is attainable only once the observation period has ended. We therefore do not present this model as an early prediction tool. But, in our view, it is still clinically relevant: during inpatient expectant management the model can be recalculated whenever new information becomes available—at each ward round or after new laboratory results—so that women whose updated predicted probability exceeds the 0.450 cut-off can be flagged for intensified maternal and fetal surveillance or reconsideration of the timing of delivery, while those below the cut-off can be reassured. In this serial-use setting the predictors are genuinely available at the moment each prediction is made, so the look-ahead structure does not invalidate the estimates; it does, however, mean that discrimination at any single early time point is unknown and is likely lower than reported here. A definitive solution requires predictors measured within a fixed window after admission—for example, a landmark analysis refitted on data available at 24 or 48 h—together with prospective temporal validation, and we consider the development and external validation of such an admission-time model the essential next step before clinical implementation.

The adjusted odds ratios for the same predictor differ between Tables 2 and 3, and the difference is expected rather than contradictory. Table 2 presents an exploratory model fitted on the raw scale and adjusted for all fourteen candidate variables, whereas Table 3 presents the final model, in which the four continuous predictors were natural-log transformed and the covariate set was reduced to the five retained variables. An OR in Table 3 therefore expresses the change in odds per e-fold (≈2.7-fold) increase in the original measurement rather than per unit, so the two sets of estimates are not on a comparable scale. For RTP, a binary variable unaffected by transformation, the attenuation from 4.30 in the exploratory model to 2.33 in the final model reflects only the change in the adjustment set. The association of RTP with HC is consistent with the central role of ascending infection in the pathogenesis of both PPROM and HC (Balogh et al., 2025; Lanzarone et al., 2025), and the high prevalence of Mycoplasma species in our cohort accords with previous reports linking genital mycoplasmas to adverse pregnancy outcomes (Noda-Nicolau et al., 2022; Jonduo et al., 2022)

Both WBC and CRP were associated with HC in univariate and multivariable analyses. These markers are well-established components of the inflammatory response and are included among the objective criteria for clinical chorioamnionitis (Megli et al., 2025; Sumino et al., 2025). Their incorporation into the model is therefore clinically plausible. The frequency of VE was positively associated with HC. This relationship may be attributed to direct transfer of pathogens *via* examination gloves, repeated mechanical disruption of the cervical mucus plug, and facilitation of bacterial translocation (Moncrieff et al., 2022). These findings align with the cohort study by Gluck et al. (2020).

LA and parity were independently associated with HC in the exploratory analysis (parity OR = 4.64, LA OR = 0.23). Our decision of leaving them out of the final model was clinical rather than statistical. Multiparity travels with a longer obstetric history—cervical trauma and ascending infection in earlier pregnancies—but in this cohort it also tracked closely with gravidity (*ρ* = 0.682) and age, so whatever the multivariable model attributes to parity is hard to separate from that history. LA poses the opposite problem: it is given selectively, most often to women whose labor is progressing well, and a protective adjusted odds ratio more plausibly marks the selection of low-risk women into analgesia than any protective effect of the analgesia itself, a direction that contradicts several reports linking epidural analgesia to intrapartum fever and infection (An et al., 2022; Lipschuetz et al., 2022). A prespecified sensitivity analysis bears this out: when both variables were added back, the five infection-related predictors retained their direction and significance and the AUC moved only from 0.901 to 0.918, with parity estimated at OR 2.94 (95% CI [1.27–6.82]) and LA at OR 0.20 (95% CI [0.08–0.51]). The parsimonious model therefore gives up almost nothing in discrimination while avoiding two coefficients that are difficult to interpret causally.

GW and duration of membrane rupture were significant in univariate analysis but lost significance after adjustment. Duration likely acts partly as a mediator: prolonged rupture increases opportunities for infection, but this risk is captured more directly by WBC, CRP, and microbiological findings. Modern PPROM management at our center—including prophylactic antibiotics, restricted digital examinations, and timely delivery—may also have attenuated the independent effect of rupture duration.

Several predictive models for chorioamnionitis have been reported. Biomarkers in amniotic fluid, including CRP, NLR, IL-1*β*, TNF-*α*, and MMP-9, have shown predictive value (Kacerovsky et al., 2024; Dong et al., 2024). Galletta et al. (2023) developed a model incorporating abdominal pain, uterine activity, fever, latency >3 days, and CRP, with an AUC of 0.726. Our model shows higher discrimination even after bootstrap correction (AUC = 0.894), but direct comparison is limited by differences in predictors, populations, and outcome ascertainment; in particular, our use of cumulative and peak in-hospital values necessarily imports more information than models restricted to admission variables.

The main limitations are the single-center retrospective design, the absence of external validation, the use of peak and cumulative predictors that are not available at admission, the modest missingness of CRP and ABT handled by complete-case analysis, the marginal events-per-variable ratio of the exploratory model (10.5, against 29.4 for the final model), and the high outcome prevalence that conditions absolute predicted probabilities and the PPV and NPV. These issues will be addressed in future prospective, multicenter validation studies and in the development of an admission-only predictor version of the model.

## Conclusions

VE, ABT, RTP, WBC, and CRP were identified as independent risk factors for HC in women with PPROM. On their basis we developed a logistic regression model for risk stratification during inpatient expectant management. The model showed good discrimination in the development sample (apparent AUC 0.901, 95% CI [0.864–0.934]), which changed little after bootstrap correction (optimism-corrected AUC 0.894), and its calibration was adequate (Hosmer–Lemeshow *P* = 0.409; Brier score 0.128). At the optimal cut-off of 0.450, sensitivity was 85.0% and specificity 79.9%. Because the predictors are cumulative or peak values recorded up to delivery, the model is intended to support decisions during hospitalization rather than at the time of admission, and it requires external validation in independent populations—together with an admission-only predictor version—before it can be considered for clinical decision-making.

##  Supplemental Information

10.7717/peerj.21929/supp-1 Document S1 TRIPOD statement

10.7717/peerj.21929/supp-2 Supplemental Information 2 STROBE checklist

10.7717/peerj.21929/supp-3 Data S1 Raw data

10.7717/peerj.21929/supp-4 Table S1 Missing values for candidate predictors (*n* = 306) Complete cases for the final five-variable model: *n* = 291 (147 events). Missing CRP values were attributable to the test not being ordered as part of routine care; the missingness fraction was low, so a complete-case analysis was retained.

10.7717/peerj.21929/supp-5 Table S2 Variance inflation factors (VIFs) for the 14 candidate predictors in Table 2 and Spearman rank correlation coefficients of parity and LA with the other candidate predictors BMI, Body mass index; GW, Gestational weeks; VE, Vaginal examinations; ABT, Antibiotic beginning time; RTP, Reproductive tract pathogens; GC, glucocorticoids; LA, Labor analgesia; T, Temperature; WBC, White blood cell; CRP, C-Reactive protein; CI, confidence interval; *, *P* < 0.05; **, *P* < 0.001. *ρ*, Spearman rank correlation coefficient.

10.7717/peerj.21929/supp-6 Table S3 Sensitivity analysis: multivariable logistic regression model including parity and LA VE, vaginal examinations; ABT, antibiotic beginning time; WBC, white blood cell count; CRP, C-reactive protein; RTP, reproductive tract pathogens; LA, labor analgesia; OR, odds ratio; CI, confidence interval.

## Additional Information and Declarations

### Data Availability

The following information was supplied regarding data availability: The raw measurements are available in the Supplemental Files.
