# Supplementary material for: Development of a predictive model for histological chorioamnionitis in patients with preterm premature rupture of membranes: a single-center retrospective cohort study
Source: PeerJ. 2026 Nov 12;14:e21929. doi: 10.7717/peerj.21929 (PMC13770420; doi:10.7717/peerj.21929)
Supplement: Supplementary file 1 [file peerj-14-21929-s001.docx]

**TRIPOD+AI Checklist**

Transparent Reporting of a multivariable prediction model for Individual Prognosis Or Diagnosis (TRIPOD+AI)

Study: Development of a prediction model for histological chorioamnionitis in women with preterm premature rupture of membranes: a single-center retrospective cohort study

This checklist is based on the TRIPOD+AI statement (Collins GS, et al. BMJ. 2024;385:e078378). Items marked D apply to model development; V to validation. This study is a development-only study.

| **Section/Item** | **Checklist item** | **Reported** | **Location in revised manuscript** |
| --- | --- | --- | --- |
| Title/abstract | 1. Identify the study as developing and/or validating a multivariable prediction model, the target population, and the outcome to be predicted. | Yes | Title; Abstract |
| Title/abstract | 2. Provide a summary of objectives, study design, setting, participants, sample size, predictors, outcome, statistical analysis, results, and conclusions. | Yes | Abstract |
| Introduction | 3a. Explain the medical context (including whether diagnostic or prognostic) and rationale for developing or validating the model, including references to existing models. | Yes | Introduction, last paragraph |
| Introduction | 3b. Specify the objectives, including whether the study describes development or validation (or both). | Yes | Abstract; Introduction, last paragraph |
| Methods | 4a. Describe the study design or source of data. | Yes | Methods, Study design and setting |
| Methods | 4b. Specify the key study dates. | Yes | Methods, Study design and setting |
| Methods | 5a. Specify key elements of the study setting including number and location of centres. | Yes | Methods, Study design and setting |
| Methods | 5b. Describe eligibility criteria for participants. | Yes | Methods, Participants |
| Methods | 5c. Give details of treatments received, if relevant. | Partial | Methods, Participants and Predictors |
| Methods | 6a. Clearly define the outcome that is predicted, including how and when assessed. | Yes | Methods, Outcome |
| Methods | 6b. Report any actions to blind assessment of the outcome. | Yes | Methods, Outcome |
| Methods | 7a. Clearly define all predictors used, including how and when measured. | Yes | Methods, Predictors and measurements |
| Methods | 7b. Report any actions to blind assessment of predictors. | Yes | Methods, Outcome; Predictors |
| Methods | 8. Explain how the study size was arrived at. | Partial | Methods, Participants; no formal sample-size calculation |
| Methods | 9. Describe how missing data were handled. | Yes | Methods, Statistical analysis |
| Methods | 10a. Describe how predictors were handled in the analyses. | Yes | Methods, Statistical analysis |
| Methods | 10b. Specify type of model, model-building procedures, and method for internal validation. | Partial | Methods, Statistical analysis; internal validation not performed |
| Methods | 10d. Specify all measures used to assess model performance. | Partial | Methods, Statistical analysis; calibration not assessed |
| Results | 13a. Describe the flow of participants through the study. | Yes | Results, Participant flow; Fig. 1; Supplementary Table S1 |
| Results | 13b. Describe participant characteristics and missing data. | Yes | Results; Table 1 |
| Results | 14a. Specify the number of participants and outcome events in each analysis. | Yes | Results; Tables 1–3 |
| Results | 14b. Report the unadjusted association between each candidate predictor and outcome. | Yes | Table 2 |
| Results | 15a. Present the full prediction model to allow predictions for individuals. | Yes | Results; Table 3 |
| Results | 15b. Explain how to use the prediction model. | Yes | Results, Final prediction model |
| Results | 16. Report performance measures with CIs. | Partial | Results; Fig. 2; AUC and CI reported; calibration absent |
| Discussion | 18. Discuss any limitations of the model and validation. | Yes | Discussion |
| Discussion | 19. Give an overall interpretation of the results. | Yes | Discussion |
| Discussion | 20. Discuss implications for practice and future research. | Yes | Discussion; Conclusions |
| Other | 21. Provide information about the availability of supplementary resources. | Yes | Supplementary Table S1; this checklist |
| Other | 22. Declare sources of funding and competing interests. | Yes | Declarations |

Note: Items 10c, 10e, 11, 12, 13c, 15b (validation-specific), and 17 are not applicable to this development-only study or were not performed.
