# Supplementary material for: Development of a predictive model for histological chorioamnionitis in patients with preterm premature rupture of membranes: a single-center retrospective cohort study
Source: PeerJ. 2026 Nov 12;14:e21929. doi: 10.7717/peerj.21929 (PMC13770420; doi:10.7717/peerj.21929)
Supplement: Supplementary file 2 [file peerj-14-21929-s004.docx]

**Supplementary Table S1. Missing values for candidate predictors (n = 306).**

| **Variable** | **Missing, n (%)** | **Available for analysis, n** |
| --- | --- | --- |
| Age | 0 (0) | 306 |
| Gravidity | 0 (0) | 306 |
| Parity | 0 (0) | 306 |
| BMI | 0 (0) | 306 |
| Gestational weeks | 0 (0) | 306 |
| Duration | 0 (0) | 306 |
| VE | 0 (0) | 306 |
| ABT | 3 (1.0) | 303 |
| RTP | 0 (0) | 306 |
| GC | 0 (0) | 306 |
| LA | 0 (0) | 306 |
| Temperature | 0 (0) | 306 |
| WBC | 0 (0) | 306 |
| CRP | 12 (3.9) | 294 |
| Outcome (HC) | 0 (0) | 306 |

Complete cases for the final five-variable model: n = 291 (147 events). Missing CRP values were attributable to the test not being ordered as part of routine care; the missingness fraction was low, so a complete-case analysis was retained.
