# Supplementary material for: Development of a predictive model for histological chorioamnionitis in patients with preterm premature rupture of membranes: a single-center retrospective cohort study
Source: PeerJ. 2026 Nov 12;14:e21929. doi: 10.7717/peerj.21929 (PMC13770420; doi:10.7717/peerj.21929)
Supplement: Supplementary file 3 [file peerj-14-21929-s005.docx]

**Supplementary Table S2a. Variance inflation factors (VIFs) for the 14 candidate predictors in Table 2.**

| **Predictor** | **VIF** |
| --- | --- |
| Age | 1.26 |
| Gravidity_bin | 1.94 |
| Parity_bin | 2.13 |
| BMI | 1.06 |
| GW | 1.81 |
| Duration | 1.58 |
| VE | 1.36 |
| ABT | 1.12 |
| RTP | 1.07 |
| GC | 1.44 |
| LA | 1.33 |
| T | 1.41 |
| WBC | 1.68 |
| CRP | 1.56 |

**Supplementary Table S2b. Spearman rank correlation coefficients of parity and LA with the other candidate predictors.**

| **Variable** | **Parity ρ** | **P** | **LA ρ** | **P** |
| --- | --- | --- | --- | --- |
| Age | 0.424 | <0.001 | -0.104 | 0.069 |
| Gravidity | 0.682 | <0.001 | -0.193 | <0.001 |
| Parity | — | — | -0.193 | <0.001 |
| BMI | 0.083 | 0.156 | 0.067 | 0.243 |
| GW | -0.113 | 0.055 | 0.278 | <0.001 |
| Duration | 0.015 | 0.802 | -0.020 | 0.724 |
| VE | -0.165 | 0.005 | 0.423 | <0.001 |
| ABT | -0.003 | 0.958 | -0.126 | 0.028 |
| RTP | -0.136 | 0.020 | -0.004 | 0.944 |
| GC | 0.099 | 0.091 | -0.179 | 0.002 |
| LA | -0.188 | 0.001 | — | — |
| T | -0.102 | 0.081 | 0.183 | 0.001 |
| WBC | -0.042 | 0.481 | -0.032 | 0.572 |
| CRP | 0.210 | <0.001 | -0.010 | 0.862 |

BMI, Body mass index; GW, Gestational weeks; VE, Vaginal examinations; ABT, Antibiotic beginning time; RTP, Reproductive tract pathogens; GC, glucocorticoids; LA, Labor analgesia; T, Temperature; WBC, White blood cell; CRP, C-Reactive protein.; CI, confidence interval; *, P<0.05; **, P<0.001. ρ = Spearman rank correlation coefficient.
