# Supplementary material for: Development of a predictive model for histological chorioamnionitis in patients with preterm premature rupture of membranes: a single-center retrospective cohort study
Source: PeerJ. 2026 Nov 12;14:e21929. doi: 10.7717/peerj.21929 (PMC13770420; doi:10.7717/peerj.21929)
Supplement: Supplementary file 4 [file peerj-14-21929-s006.docx]

**Supplementary Table S3. Sensitivity analysis: multivariable logistic regression model including parity and LA**

| **Variable** | **B** | **OR** | **95% CI** | **P** |
| --- | --- | --- | --- | --- |
| Intercept | -13.257 | 0.000 | - | <0.001 |
| ln(VE) | 2.761 | 15.818 | 6.580–38.027 | <0.001 |
| ln(ABT) | 1.066 | 2.904 | 1.778–4.743 | <0.001 |
| ln(WBC) | 2.690 | 14.735 | 3.412–63.640 | <0.001 |
| ln(CRP) | 0.765 | 2.150 | 1.453–3.180 | <0.001 |
| RTP | 1.145 | 3.141 | 1.495–6.599 | 0.003 |
| Parity | 1.078 | 2.940 | 1.266–6.824 | 0.012 |
| LA | -1.603 | 0.201 | 0.080–0.507 | <0.001 |

VE, vaginal examinations; ABT, antibiotic beginning time; WBC, white blood cell count; CRP, C-reactive protein; RTP, reproductive tract pathogens; LA, labor analgesia; OR, odds ratio; CI, confidence interval.
