# Supplementary material for: Development of a predictive model for histological chorioamnionitis in patients with preterm premature rupture of membranes: a single-center retrospective cohort study
Source: PeerJ. 2026 Nov 12;14:e21929. doi: 10.7717/peerj.21929 (PMC13770420; doi:10.7717/peerj.21929)
Supplement: Supplementary file 5 [file peerj-14-21929-s002.doc]

STROBE Statement—Checklist of items that should be included in reports of ***cohort studies***

|  | Item No | Recommendation | Page No. | Relevant text from manuscript |
| --- | --- | --- | --- | --- |
| **Title and abstract** | 1 | (*a*) Indicate the study’s design with a commonly used term in the title or the abstract | 1 | Line 3-4, Line 24 |
|  |  | (*b*) Provide in the abstract an informative and balanced summary of what was done and what was found | 1 | Line 20-43 |
| Introduction | | |  |  |
| Background/rationale | 2 | Explain the scientific background and rationale for the investigation being reported | 1 | Line 45-72 |
| Objectives | 3 | State specific objectives, including any prespecified hypotheses |  | Line 73-77 |
| Methods | | |  |  |
| Study design | 4 | Present key elements of study design early in the paper | 3 | Line 80 |
| Setting | 5 | Describe the setting, locations, and relevant dates, including periods of recruitment, exposure, follow-up, and data collection | 3 | Line 80-83 |
| Participants | 6 | (*a*) Give the eligibility criteria, and the sources and methods of selection of participants. Describe methods of follow-up | 3 | Line 88-97 |
|  |  | (*b*) For matched studies, give matching criteria and number of exposed and unexposed |  | N/A |
| Variables | 7 | Clearly define all outcomes, exposures, predictors, potential confounders, and effect modifiers. Give diagnostic criteria, if applicable | 3 | Line99-107 |
| Data sources/ measurement | 8* | For each variable of interest, give sources of data and details of methods of assessment (measurement). Describe comparability of assessment methods if there is more than one group | 3-4 | Line109-125 |
| Bias | 9 | Describe any efforts to address potential sources of bias | 3 | consecutive enrollment to prevent selection bias; use of gold-standard pathological diagnosis and pre-collected structured data to reduce information bias |
| Study size | 10 | Explain how the study size was arrived at | 3 | The sample size of this retrospective cohort study was determined by the consecutive enrollment of all eligible patients with premature rupture of membranes at our center between December 2022 and October 2025, resulting in a final cohort of 308 participants. |
| Quantitative variables | 11 | Explain how quantitative variables were handled in the analyses. If applicable, describe which groupings were chosen and why |  | Line 147-149 |
| Statistical methods | 12 | (*a*) Describe all statistical methods, including those used to control for confounding | 4 | Line 139-150 |
|  |  | (*b*) Describe any methods used to examine subgroups and interactions |  | N/A |
|  |  | (*c*) Explain how missing data were addressed | 5 | Line165-172 |
|  |  | (*d*) If applicable, explain how loss to follow-up was addressed |  | N/A |
|  |  | (*e*) Describe any sensitivity analyses |  | Line 151-156 |
| Results | | |  |  |
| Participants | 13* | (a) Report numbers of individuals at each stage of study—eg numbers potentially eligible, examined for eligibility, confirmed eligible, included in the study, completing follow-up, and analysed |  | Fig 1 |
|  |  | (b) Give reasons for non-participation at each stage |  | Fig 1 |
|  |  | (c) Consider use of a flow diagram |  | Fig 1 |
| Descriptive data | 14* | (a) Give characteristics of study participants (eg demographic, clinical, social) and information on exposures and potential confounders | 3 | Line165-172 |
|  |  | (b) Indicate number of participants with missing data for each variable of interest | 5 | Line191-194 |
|  |  | (c) Summarise follow-up time (eg, average and total amount) | 3 | N/A |
| Outcome data | 15* | Report numbers of outcome events or summary measures over time |  | Line163-164 |
| Main results | 16 | (*a*) Give unadjusted estimates and, if applicable, confounder-adjusted estimates and their precision (eg, 95% confidence interval). Make clear which confounders were adjusted for and why they were included | 4 | Line185-188 |
|  |  | (*b*) Report category boundaries when continuous variables were categorized |  | Table 1 |
|  |  | (*c*) If relevant, consider translating estimates of relative risk into absolute risk for a meaningful time period |  | N/A |
| Other analyses | 17 | Report other analyses done—eg analyses of subgroups and interactions, and sensitivity analyses | 4 | Line203-209 |
| Discussion | | |  |  |
| Key results | 18 | Summarise key results with reference to study objectives | 5 | Line 211-216 |
| Limitations | 19 | Discuss limitations of the study, taking into account sources of potential bias or imprecision. Discuss both direction and magnitude of any potential bias | 7 | Line 281-287 |
| Interpretation | 20 | Give a cautious overall interpretation of results considering objectives, limitations, multiplicity of analyses, results from similar studies, and other relevant evidence | 5-7 | Line 217-280 |
| Generalisability | 21 | Discuss the generalisability (external validity) of the study results | 7 | Line 250-258 |
| Other information | | |  |  |
| Funding | 22 | Give the source of funding and the role of the funders for the present study and, if applicable, for the original study on which the present article is based | 8 | Line 315-316 |

*Give information separately for exposed and unexposed groups.

**Note:** An Explanation and Elaboration article discusses each checklist item and gives methodological background and published examples of transparent reporting. The STROBE checklist is best used in conjunction with this article (freely available on the Web sites of PLoS Medicine at http://www.plosmedicine.org/, Annals of Internal Medicine at http://www.annals.org/, and Epidemiology at http://www.epidem.com/). Information on the STROBE Initiative is available at http://www.strobe-statement.org.
